# Sex-based variations in breath-holding: oxygen storage and diving response among non-divers

**DOI:** 10.3389/fphys.2024.1515232

**Published:** 2025-01-13

**Authors:** Frank Pernett, Erika Schagatay, Pontus Holmström

**Affiliations:** ^1^ Department of Health Sciences, Environmental Physiology Group, Mid Sweden University, Östersund, Sweden; ^2^ Department of Health Sciences, Swedish Winter Sports Research Centre, Mid Sweden University, Östersund, Sweden

**Keywords:** apnea, diving response, freediving, hypoxia, sex differences, splenic contraction, hypoxia tolerance

## Abstract

Breath-hold diving performances are typically better in men than in women. However, it is still being determined if there are differences in the physiological responses to breath-holding between the sexes. We conducted a study comparing the maximum breath-hold duration, heart rate (HR) reduction, peripheral oxygen saturation (SpO_2_), and spleen volume and contraction in 37 men and 44 women, all of whom had no prior breath-holding experience. They performed two dry apneas separated by 2 min; the first was limited to 60 s, followed by a maximal effort apnea. HR and SpO_2_ were measured continuously. Spleen diameters were measured via ultrasonography before and immediately following each apnea. The maximal apneic duration was longer in men (78 ± 19 s) compared with women (61 ± 18 s, p < 0.001), while the HR reduction was similar (women: 16% ± 19% versus men: 16% ± 17%, p = 0.973). The absolute splenic contraction was greater in men (59 ± 56 mL) compared with women (35 ± 28 mL, p < 0.001) in the first apnea, while the relative contraction was similar (women: 21% ± 17% versus men: 23% ± 13%, p = 0.528). In addition, the lowest SpO_2_ during the maximal apnea was similar between sexes (women: 93.3% ± 4.4%; men: 91.9% ± 4.3%, p = 0.161). We conclude that men have larger spleen size and contraction, lung size, and maximal apneic duration than women. The cardiovascular diving response is similar between sexes for those inexperienced with apneic diving. The longer breath-hold duration in men may be partly due to greater oxygen storage capacity, which results from larger vital capacity and greater spleen size and contraction.

## 1 Introduction

Several anthropometric and physiological differences between sexes can significantly influence how individuals respond to various stressors. Some differences between sexes are mainly a result of general size differences; however, differences between sexes may also result from cultural factors (e.g., exposure and training). In short, women typically have higher heart rate (HR) at rest (Legato and Leghe, 2010), smaller height-adjusted lung volumes (Schwartz et al., 1988), and lower hemoglobin concentration (Hawkins et al., 1954; Murphy, 2014) and plasma volume (Gandhi et al., 2004), compared with men. During periods of hypoxia or apnea, the capacity of the body to retain and use oxygen (O_2_) is critical. Considering that the biggest sources of oxygen are the lungs and blood, the mentioned physiological traits could influence the amount of O_2_ storage between sexes, raising an important question about whether these sex-based differences become even more pronounced in environments where oxygen supply is limited.

Several sports are performed with limited O_2_ supply, including high-altitude climbing, parachuting, synchronized swimming, underwater hockey, and breath-hold diving. In all these sports, performance is, to some extent, limited by hypoxia and asphyxia tolerance. Few sex differences related to hypoxia tolerance have been identified, e.g., at high altitude. There is evidence that women are at greater risk of developing acute mountain sickness during ascent to high altitude. However, the underlying cause of this difference is unclear (Richalet et al., 2012; Hou et al., 2019). Whether such physiological sex differences exist in breath-hold diving (also known as apneic diving or freediving) and consequently influence tolerance to apnea-induced hypoxia is less clear. Breath-hold diving does not involve any respiratory support; therefore, it depends entirely on the individual ability to sustain a breath-hold. Apneic duration is determined by total body O_2_ storage, rate of metabolism, i.e., O_2_ consumption, tolerance to asphyxia, and, to some extent, psychological endurance (Schagatay, 2009). While controlled research observations display no sex differences in apneic durations (Jay and White, 2006; Cherouveim et al., 2013), the current world records in competitive freediving are greater in men compared with women in all disciplines (AIDA, 2024; CMAS, 2024). Recent research observed greater static apnea duration in men than women, whereby the duration correlated positively with the vital capacity (Peng et al., 2021). Whether there exists a physiological basis underpinning greater apneic duration in men is unclear; plausibly, the differences could result from increased lung O_2_ stores, recruitment- or training opportunities differences.

Hypoxia tolerance is typically characterized as the ability to tolerate critically low arterial pressure of O_2_ (PaO_2_), facilitated by integrative physiological mechanisms, capable of maintaining O_2_ homeostasis (Hochachka, 1998; Bailey et al., 2017). The human diving response involves bradycardia and reduced cardiac output (CO), mediated by parasympathetic activation, alongside peripheral vasoconstriction, which redistributes blood flow and is mediated by sympathetic activation (Gooden, 1994; Panneton, 2013). This response has an O_2_-conserving effect (Schagatay and Andersson, 1998; Andersson et al., 2002), reducing O_2_ consumption in peripheral tissues through decreased blood flow (Joulia et al., 2009), as well as lowering the O_2_ demand of the myocardium (Schagatay, 2009). By conserving O2, the diving response helps protect against brain hypoxia and prolongs apnea duration (Schagatay and Andersson, 1998). During apneic diving, the cardiovascular diving response and the splenic contraction are considered vital responses that preserve the arterial O_2_ content (CaO_2_; Bouten et al., 2019; Schagatay et al., 2001), thereby acting protectively to prevent severe hypoxia.

The diving response is initiated by cessation of breathing and reinforced by stimulation of facial cold receptors, e.g., via cold water immersion (Schuitema and Holm, 1988; Schagatay and Andersson, 1998). Trained and Indigenous freedivers display a more pronounced diving response and a more efficient O_2_ conserving mechanism compared with non-divers (Schagatay and Andersson, 1998; Tocco et al., 2012; Costalat et al., 2017), confirming the benefit of a pronounced diving response. Consequently, maximal apneic duration relates to the magnitude of the diving response (Schagatay and Andersson, 1998). Previous observations on sex differences in the cardiovascular diving response are conflicting. One study found no sex difference in HR reduction in non-divers (Cherouveim et al., 2013), while another study found no difference between sexes in both breath-hold divers and non-divers (Peng et al., 2021). On the other hand, another study found a more pronounced HR reduction in men during 40 s apneas with face immersion compared to women, whereas HR reductions were similar during dry apneas (Tocco et al., 2012). Furthermore, studies by Peng et al. (2021) on face immersion and Tocco et al. (2012) on dry apneas indicate comparable responses in stroke volume and CO across sexes. This complicates the interpretation of sex differences in the diving response. Therefore, current observations regarding these differences are ambiguous and require further investigation.

Another protective mechanism against hypoxia during apneic diving is the splenic contraction, which results in a transient increase in [Hb] by ∼4% (Schagatay et al., 2007; Bouten et al., 2019; Elia et al., 2019), which is returned to baseline values within ∼10 min after apnea (Schagatay et al., 2005). The [Hb] elevation response is absent in splenectomized individuals (Schagatay et al., 2001; Baković et al., 2003; 2005). The splenic contraction is stimulated by hypoxia-induced sympathetic nervous system activation (Baković et al., 2003; Richardson et al., 2008; Lodin-Sundström and Schagatay, 2010; Pernett et al., 2021). Additionally, the response has been observed during apneic diving (Schagatay et al., 2005; Holmström et al., 2019), exercise (Laub et al., 1993; Stewart et al., 2003), and at high altitude (Schagatay et al., 2020b; Holmström et al., 2021). Competitive freedivers, high-altitude climbers, and indigenous highlanders have larger spleens and greater splenic contraction compared with controls (Schagatay et al., 2012; Schagatay et al., 2020a; Holmström et al., 2020), supporting the functional benefit of greater splenic contraction during hypoxia exposure. However, current reports on differences between sexes are not as compelling. While most observations suggest that men have larger spleens compared to women (Hosey et al., 2006; Ehimwenma and Tagbo, 2011; Chow et al., 2016), it has also been reported that no difference exists between sexes (Prassopoulos et al., 1997). Furthermore, no previous research has examined sex differences in the splenic response, i.e., the magnitude of the splenic contraction.

Accordingly, it is unclear whether women and men have different physiological responses to apneic diving, i.e., the cardiovascular diving response and splenic contraction. We, therefore, aimed to compare the HR reduction and spleen size and contraction between men and women previously inexperienced with apneic diving. We hypothesized that the diving response would be similar between sexes while splenic contraction would be greater in men.

## 2 Methods

### 2.1 Recruitment and sample size

Eighty-one healthy participants (Table 1) volunteered for the investigation and were all recruited via convenience sample through verbal communication. Investigating non-divers is vital to avoid confounding training-induced effects on the studied responses. Therefore, we recruited participants previously not experienced in breath-holding. Participants were divided based on sex: 44 women and 37 men (Table 1). These group sizes met the minimum sample size criteria required for the statistical test, ensuring an adequate number of participants in each group of the independent variable, as recommended by Hair et al. (2006). In addition, to reduce the risk of an underpowered result, *a priori* sample size calculation was made in G*power (version 3.0.10), wherein a strong effect size was expected alongside a significant alpha level of 0.01 and a power of 0.9, which yielded a sample size of >35 of each independent group. A portion of the study sample from both sexes was tested in 2016, while the main sample was assessed in 2018. All measurements were conducted at altitudes below 1,500 m. After receiving detailed written and oral information about the procedures, participants gave their written informed consent to participate. This study abided by the Declaration of Helsinki involving human participants, and the regional committee for medical and health research ethics in Umeå had approved the study protocol.

**TABLE 1:** Participant demographics.

	Women	Men	p-value	ES	95% CI
n	44	37			
Age	35 ± 12	35 ± 15	0.905	0.00	0.00–0.03
Height (cm)	166.6 ± 6.6	180.2 ± 7.7	<0.001	0.48	0.32–0.56
Body mass (kg)	65.8 ± 10.5	78.2 ± 10.9	<0.001	0.26	0.10–0.40
VC (L)	4.1 ± 0.5	5.6 ± 0.9	<0.001	0.50	0.33–0.60
Spleen (mL)	151 ± 50	253 ± 80	<0.001	0.39	0.22–0.52

Mean ± SD. ES, effect size; CI, confidence interval; cm, centimeters; kg, kilograms; L, liters; mL, milliliters; n, number of participants; VC, vital capacity; Spleen, spleen volume. Age expressed in years.

### 2.2 Study protocol

A dry apnea test was used to induce hypoxia and subsequent physiological responses. Dry apneas were chosen for logistic reasons and have been shown to induce both the diving response and splenic contraction in non-divers (Andersson et al., 2002; Schagatay et al., 2007; Holmstrom et al., 2019). The test has been developed to analyze physiological responses induced by apneas of the same duration and those of maximal effort (Holmström et al., 2019). Participants arrived at the laboratory in a fed and rested state and were instructed to abstain from consuming caloric or caffeine-containing beverages for 1 h before testing. They abstained from alcohol and tobacco for at least 24 h before testing. Height (cm) and body mass (kg) were measured, after which they were seated to rest on a chair where the apnea test was conducted. After a 2 min resting baseline, VC (L) was recorded in duplicate (Vitalograph Ltd., Compact II, Buckingham England), and the larger volume was used for analysis. Following a minimum of 10 min of rest, a 2 min countdown for the apnea test started.

### 2.3 Apnea test

**FIGURE 1:**
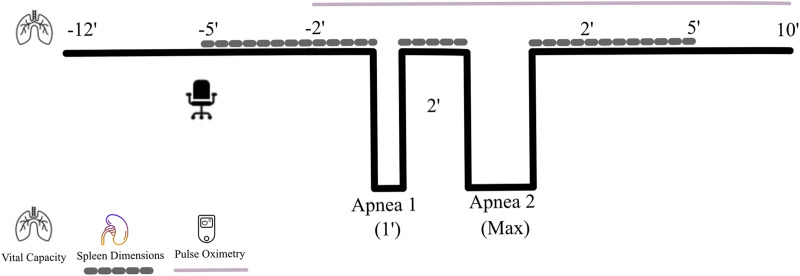
Protocol for the apnea test. During the seated rest, the last 5 min period was used to collect splenic measurements every minute (dotted line) for baseline assessments, after which a voluntary breath-hold of an intended 1 min duration (Apnea 1) was completed. Immediately after its termination, spleen dimensions were measured, after which the maximal breath-hold (Apnea 2) started. Immediately after termination of the maximal apnea, splenic measurements were collected again every minute, which continued during 5 min of seated rest. Heart rate (HR) and peripheral oxygen saturation (SpO_2_) were measured continuously (continuous line) from 2 min before the first breath-hold until 10 min following the last breath-hold.

The apnea protocol consisted of two dry apneas (without facial immersion) separated by 2 minutes of rest (Figure 1), the first limited to 1 min duration (time-limited apnea; Apnea 1) and the second of maximal voluntary duration (Apnea 2). One minute before each apnea, the participant was notified about the remaining time, and 30 s before, a nose clip was attached. Apneas were conducted in the sitting position, starting after a deep but not maximal inspiration, an instruction that typically results in an inhalation equivalent to 80%–85% of the vital capacity (Andersson and Schagatay, 1997). Participants were told to relax the chest and refrain from swallowing or exhaling during apneas. As a precautionary measure to prevent the risk of hypoxic syncope, participants would be required to terminate the apnea and resume breathing if SpO2 fell below 70%. This scenario never occurred. Upon reaching their maximal duration for voluntary breath-hold duration, the participants terminated the apnea by resuming normal breathing and removing the nose clip. Time cues were given in the first apnea, intended to reach 1 minute, while no feedback was given on apneic time in the maximal attempt.

### 2.4 Cardiovascular measurements

To continuously record HR and SpO_2_, a pulse oximeter (Medair Lifesense LS1-9R, Medair AB, Delsbo, Sweden) was used, which was attached with sensors to the tip of the index finger. HR and SpO_2_ were measured 2 minutes before the first apnea until 10 min after the last apnea (Figure 1). Data were stored via a memory unit (Trendsense, Nonin Medical Inc., Medair AB, Hudiksvall, Sweden) for later analysis.

### 2.5 Spleen ultrasound measurements

The spleen size was measured from the dorsal side of the body via ultrasonic imaging (M-Turbo Ultrasound system, FUJIFILM Sonosite Inc., Bothell, WA United States) by an experienced sonographer (PH). Following 5 min of pre-collection measurements aimed to identify the spleen and its associated landmarks in both the sagittal and the transverse plane, measurements of spleen size (cm) were collected each minute for 5 min before the first apnea (baseline), between apneas (spleen volume reduction) and immediately following termination of the last apnea (spleen volume reduction) for 5 min (recovery, Figure 1). At each minute of data collection, three maximal splenic dimensions (length, thickness, and width) were measured from still images. A challenge with using ultrasonography to determine spleen volume is the potential for measurement error, which can arise from the subjective identification of anatomical landmarks and biological variability due to high pulsatile changes (Cesta, 2006). However, we recently demonstrated that our method has high reliability, as indicated by a low coefficient of variation (2.98% ± 0.1%) and a strong intraclass correlation coefficient (0.970, p < 0.001) between repeated tests (Holmström et al., 2020). Moreover, spleen volumes measured by ultrasound show strong correlations with actual spleen volumes obtained from cadaveric measurements (Loftus et al., 1999) and from computed tomography scan measurements (Lamb et al., 2002).

### 2.6 Analysis

Baseline HR was defined as the mean HR from 90 to 30 s before the apnea (Schagatay, 1996; Holmström et al., 2019). The magnitude of the diving response was quantified by the apnea-induced reduction in HR as a percentage of baseline HR. We calculated the maximal apnea-induced HR reduction, where the lowest apneic HR was defined, thus eliminating the initial tachycardia (Andersson and Schagatay, 1998). As diving response arise around the first 30 s of apnea (Andersson and Schagatay, 1998), the data analysis was conducted even if the duration of apnea 1 was less than 60 s.

Baseline SpO_2_ was defined as the mean SpO_2_ from 90 to 30 s before the apnea. Apnea-induced O_2_ desaturation was measured by calculating the percentage change between the baseline SpO_2_ and the lowest SpO_2_ observed from the start of the apnea until 30 s after it ended. This accounts for the circulatory delay from the lung to the finger.

Measurements of the maximal splenic length (L), thickness (T), and width (W) were used to calculate spleen volume (Vspleen) according to the Pilström equation (Schagatay et al., 2005)

$$Vspleen=\left(\frac{L_{\pi }\left(W\left(T-T^{2}\right)\right)}{3}\right)$$

The formula describes the difference between two ellipsoids and has previously been used in similar experiments involving spleen volume assessments in association with apnea and exercise (Engan et al., 2014; Holmström et al., 2020) and has recently been associated with high reliability between repeated measurements (Holmstrom et al., 2020). Individual baseline (resting) spleen volume was obtained from the 5 min before the apnea by averaging the two consecutive maximal measurement values. This method was implemented to limit any influence of measurement error that may occur due to pulsatile changes in spleen volume, anticipatory volume changes, and/or minor changes in probe placement (Schagatay et al., 2012; Holmström et al., 2019). The individual splenic contraction was then calculated as an absolute (mL) and relative (%) change in the volume from baseline to the first measurement immediately after each apnea.

To determine possible sex differences in splenic volume contraction and VC, these values were adjusted to each participant’s height (mL·cm^−1^ and L·cm^−1^, respectively) and presented alongside absolute values.

### 2.7 Statistical analysis

Data are expressed as mean ± standard deviation (SD) unless otherwise stated and were statistically analyzed using IBM SPSS 24.0 for Windows (SPSS Inc., Chicago, IL.). Shapiro-Wilks test (p > 0.05) was used to assess if data were normally distributed. To assess the assumption of homogeneity of variance, Leven’s test of equality was conducted (p > 0.05), and homogeneity of variance-covariance was determined with Box`s M test of equality (p > 0.001). To determine whether women and men differed statistically on the dependent variables, a one-way multivariate analysis of variance (MANOVA) was conducted. The selected dependent variables were included in the model based on their individual relevance to apneic diving. In the analysis, the MANOVA assessed differences between the independent groups (women and men) by evaluating a linear combination of the dependent variables, combined into a single multivariate test statistic, to identify overall group differences. A univariate one-way analysis of variance (ANOVA) was performed to assess which of the dependent variables was statistically different, and a *post hoc* test with Bonferroni adjustment was run to follow up on any significant differences. The interaction effects between men and women on spleen volume change were assessed using a 2 × 4 mixed ANOVA with Bonferroni adjustments for multiple comparisons. Assessment of within-group differences on relevant dependent variables was conducted using repeated measures one-way ANOVA with Bonferroni adjustments for multiple comparisons. Height was used as a covariate in these tests. Associations between selected dependent variables were assessed using Pearson’s product-moment correlation coefficient (r). Effect sizes were estimated by the partial eta squared ( $\eta_{p}^{2}$ ) and are presented with a 95% confidence interval (CI). An effect size of 0.01–0.05 was considered small, from 0.06–0.13 was considered medium, and from and above 0.14 was considered large (Lakens, 2013).

## 3 Results

**FIGURE 2:**
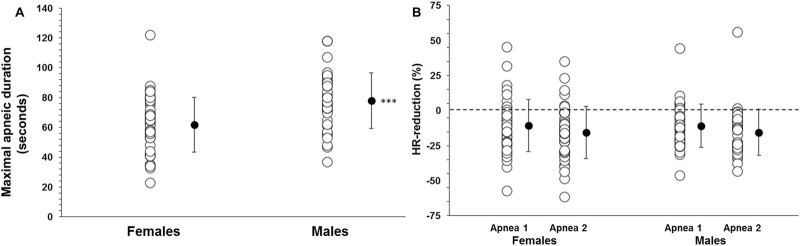
**(A)** Apnea 2 duration (seconds) in women (N = 44) and men (N = 37) and **(B)** diving response, quantified by heart rate (HR) reduction (%), during Apnea 1 and Apnea 2 for women and men. *** indicates p < 0.001 between sexes. Black dots indicate mean ± SD.

Participant’s demographic data are shown in Table 1. The MANOVA revealed a significant main effect between sexes on the combined dependent variables: maximal apneic duration, spleen size, splenic contraction, VC and HR reduction (F (5, 75) = 26.336, p < 0.001; Wilks' Λ = 0.363; η_p^∧2^ = 0.637, 95% CI [0.5–0.7]). Apnea 2, with 69 ± 20 s, was longer than Apnea 1, with 54 ± 10 s in the whole group (p < 0.001, η_p^∧2^ = 0.567, 95% CI [0.4–0.7]). Apnea 2 duration was longer in men at 78 ± 19 s compared with 62 ± 19 s in women (p < 0.001, η_p^∧2^ = 0.151, 95% CI [0.03–0.29]; Figure 2A).

### 3.1 Diving response

The resting HR was higher in women at 83 ± 16 bpm than in men at 73 ± 14 bpm (p = 0.005). Additionally, the lowest HR during Apnea 1 was higher in women (73 ± 16 bpm) than in men (65 ± 14 bpm; p = 0.016). Similarly, the lowest HR during Apnea 2 was also higher in women (68 ± 14 bpm) than in men (61 ± 12 bpm; p = 0.014). However, the HR reduction during Apnea 1 was similar between women and men (10 ± 16 bpm (11% ± 19%) and 9 ± 13 bpm (11% ± 16%), respectively; p = 0.658), Figure 2B. During Apnea 2, the HR reduction was also similar between women and men (15 ± 17 bpm (16% ± 19%) and 12 ± 12 bpm (16% ± 17%), respectively; p = 0.490), Figure 2B. The reduction in HR was greater in both men and women during Apnea 2 compared to Apnea 1 (p < 0.001, η_p^∧2^ = 0.29, 95% CI [0.14–0.43]).

There was no correlation between HR reduction and the longest apneic duration for either men or women (p = 0.836). However, for men, the HR reduction was positively correlated with spleen volume (r = 0.454, p = 0.005, Figure 6B), whereas this correlation was not present for women (r = 0.204, p = 0.183). In addition, HR reduction during Apnea 1 was positively correlated with HR reduction during Apnea 2 for women (r = 0.920, p < 0.001) and men (r = 0.889, p < 0.001).

### 3.2 Spleen volume and contraction

**FIGURE 3:**
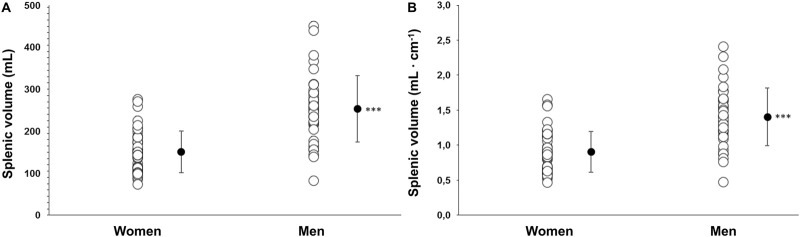
**(A)** Baseline (resting) spleen volume as an absolute value (mL) in women (N = 44) and men (N = 37) and **(B)** baseline spleen volume scaled to each participant’s height (mL·cm^−1^). *** indicates p < 0.001 between sexes. Black dots indicate mean ± SD.

**FIGURE 4:**
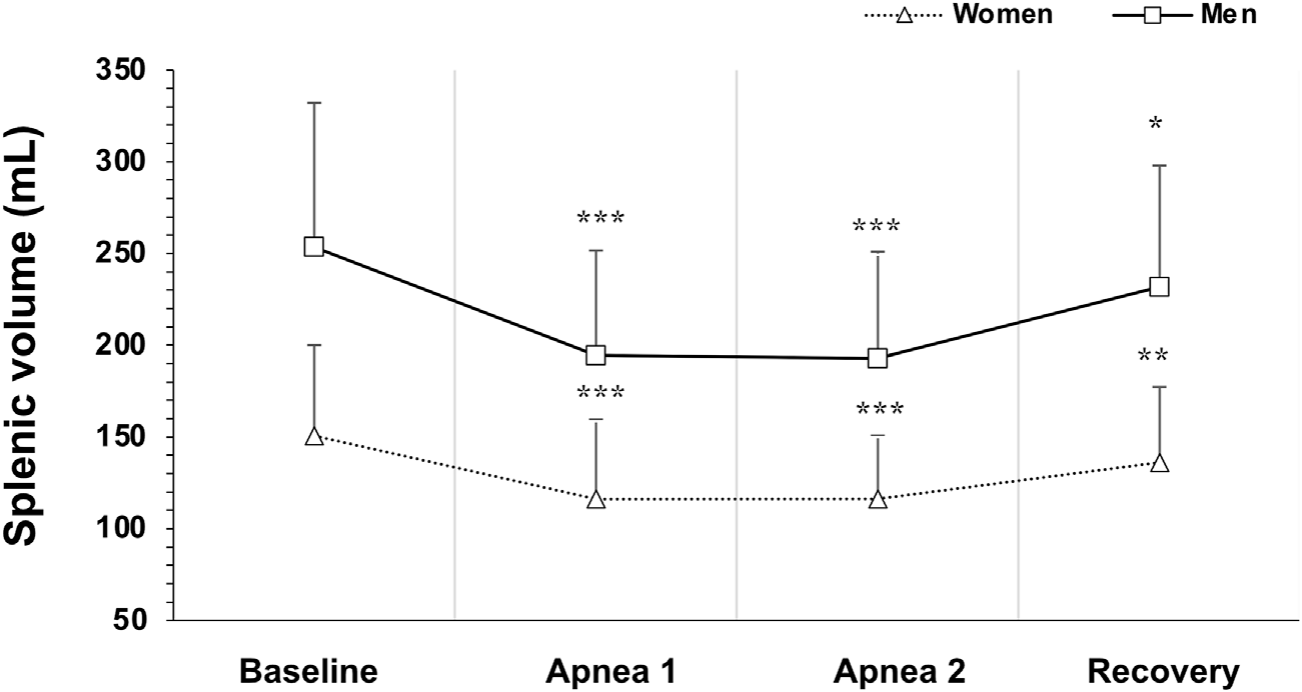
Mean ± SD Splenic volume during rest, during the two apneas, and after 5 min recovery for women (N = 44) and men (N = 37). * Indicates p < 0.05, ** indicates p < 0.01 and *** indicates p < 0.001 for within group differences from baseline volume.

**FIGURE 5:**
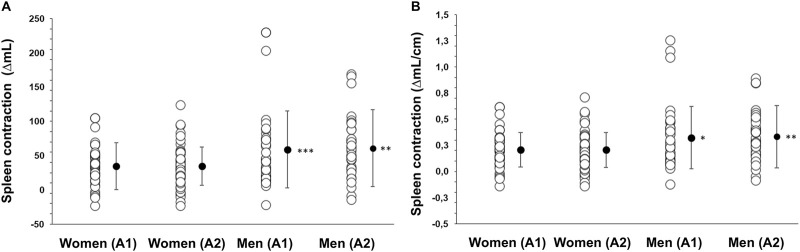
**(A)** Splenic contraction quantified as the change from baseline volume (ΔmL) with the lowest volume after Apnea 1 (A1) and Apnea 2 (A2) in mL in women (N = 44) and men (N = 37) and **(B)** splenic contraction during Apnea 1 (A1) and Apnea 2 (A2) as a relative measure adjusted to height (ΔmL·cm^−1^). * Indicates p < 0.05, ** indicates p < 0.01 and *** indicates p < 0.001 between sexes. Black dots indicate mean ± SD.

Baseline spleen volume was larger in men at 253 ± 79 mL (1.40 ± 0.4 mL·cm^−1^) compared with women at 150 ± 49 mL (0.90 ± 0.3 mL·cm^−1^), with a mean difference of 103 mL (95% CI 73–131, p < 0.001; Figure 3). Splenic contraction occurred during both apneas for both men (p < 0.001; Figure 4) and women (p < 0.001; Figure 4). However, there was an interaction effect between men and women on splenic volume change (*F* (3, 201.6) = 4.565, p = 0.004, $\eta_{p}^{2}$ = 0.06, 95% CI [0.00–0.13]; Figure 4), showing that splenic volume does not change equally between men and women in response to apnea. Splenic contraction during Apnea 1 was greater in magnitude in men at 59 ± 56 mL (0.32 ± 0.3 mL·cm^−1^) compared with women at 35 ± 28 mL (0.21 ± 0.2 mL·cm^−1^, p > 0.05; Figure 5). Similarly, splenic contraction induced by the maximal apnea was also larger in men at 60 ± 42 mL (0.33 ± 0.2 mL·cm^−1^) compared with women at 34 ± 31 mL (0.20 ± 0.18 mL·cm^−1^, p > 0.01; Figure 5).

**FIGURE 6:**
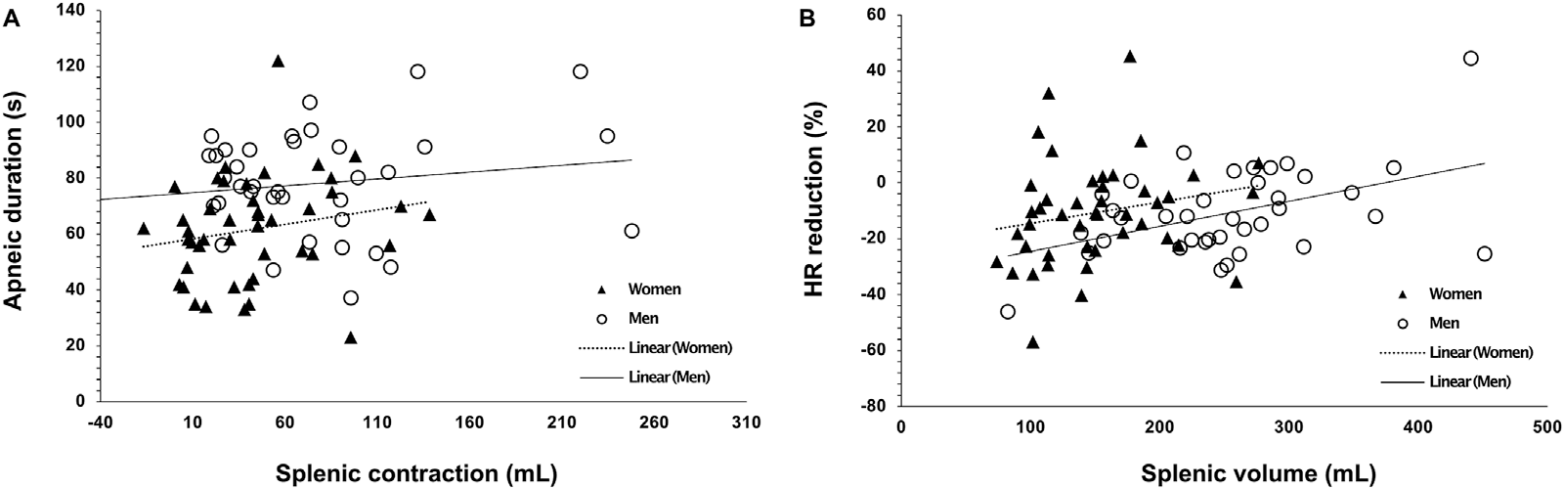
Correlation plots of **(A)** splenic contraction (mL) during Apnea 2 with maximal apneic duration (seconds; r = 0.273, p = 0.014), and **(B)** baseline (resting) splenic volume (mL) with the diving response, quantified by heart rate (HR) reduction (%), induced by the time-limited apnea (r = 0.248, p = 0.026). Closed triangles indicate women’s data points (N = 44), and open circles indicate men’s data points (N = 37).

However, the magnitude of the contraction measured as a percentage change during Apnea 1 and Apnea 2 was similar for both men (A1: 21%, A2: 23%) and women (A1: 22%, A2: 21%, p = 0.343). After 5 min of recovery, spleen volume had not returned to baseline values for either men (p = 0.041; Figure 4) or women (p = 0.005; Figure 4). There was no correlation between splenic contraction and maximal apneic duration for men (r = 0.157, p > 0.05) or women (r = 0.199, p > 0.05, Figure 6A) separately. When sexes were pooled, however, the splenic contraction correlated positively, albeit weakly, with the apneic duration (r = 0.273, p < 0.014).

### 3.3 Vital capacity

**FIGURE 7:**
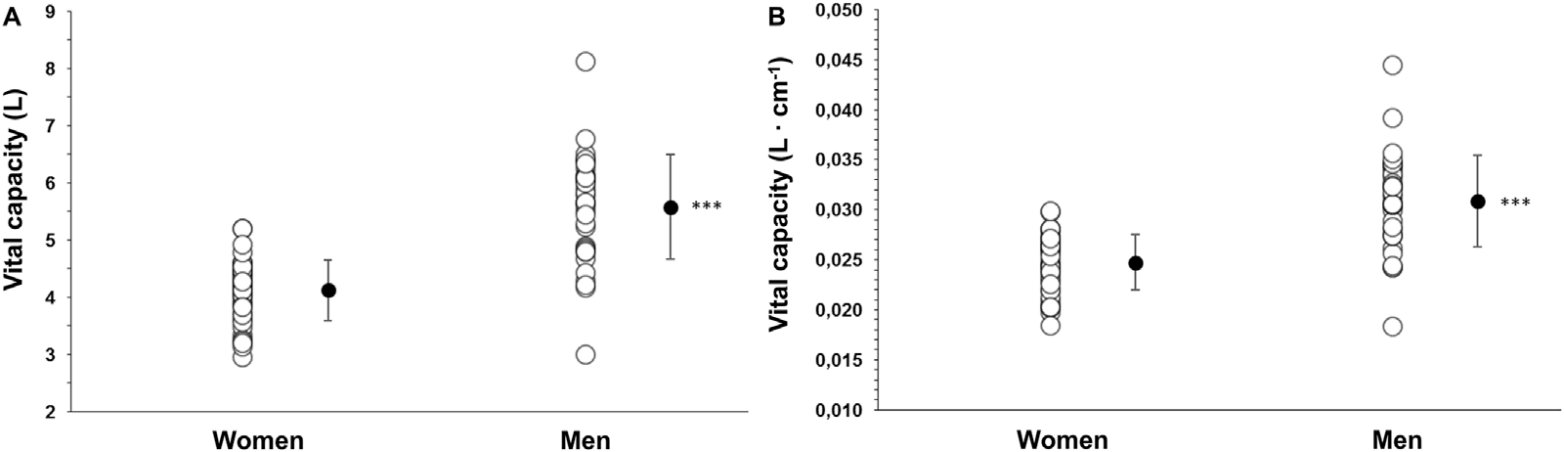
**(A)** Vital capacity (VC) as an absolute measure (L) in women (N = 44) and men (N = 37) and **(B)** VC as a measure adjusted to height (L·cm^−1^). *** indicates p < 0.001 between sexes. Black dots indicate mean ± SD.

VC was larger in men at 5.6 ± 0.9 L (0.03 ± 0.005 L·cm^−1^) compared with women at 4.1 ± 0.5 (0.02 ± 0.003 L·cm^−1^; p < 0.001, η_p^∧2^ = 0.49, 95% CI [0.33–0.60]), with a mean difference of 1.4 L (95% CI 1.1–1.7; Figure 7). VC was positively correlated with maximal apneic duration for men (r = 0.400, p = 0.018) but not for women (r = 0.017, p = 0.911).

### 3.4 Oxygen saturation

The lowest SpO_2_ during Apnea 1 was comparable between women and men, at 93.9% ± 2.7% for women and 93.6% ± 3.2% for men (p = 0.568). Similarly, during Apnea 2, the lowest SpO_2_ was similar for both sexes (women: 93.3% ± 4.4%; men: 91.9% ± 4.3%, p = 0.161). The decrease in SpO_2_ from baseline was similar between sexes during Apnea 1 (women: 3.4% ± 2.3%; men: 2.7% ± 2.3%, p = 0.215) and Apnea 2 (women: 4.1% ± 4.0%; men: 4.4% ± 4.3%, p = 0.690).

**FIGURE 8:**
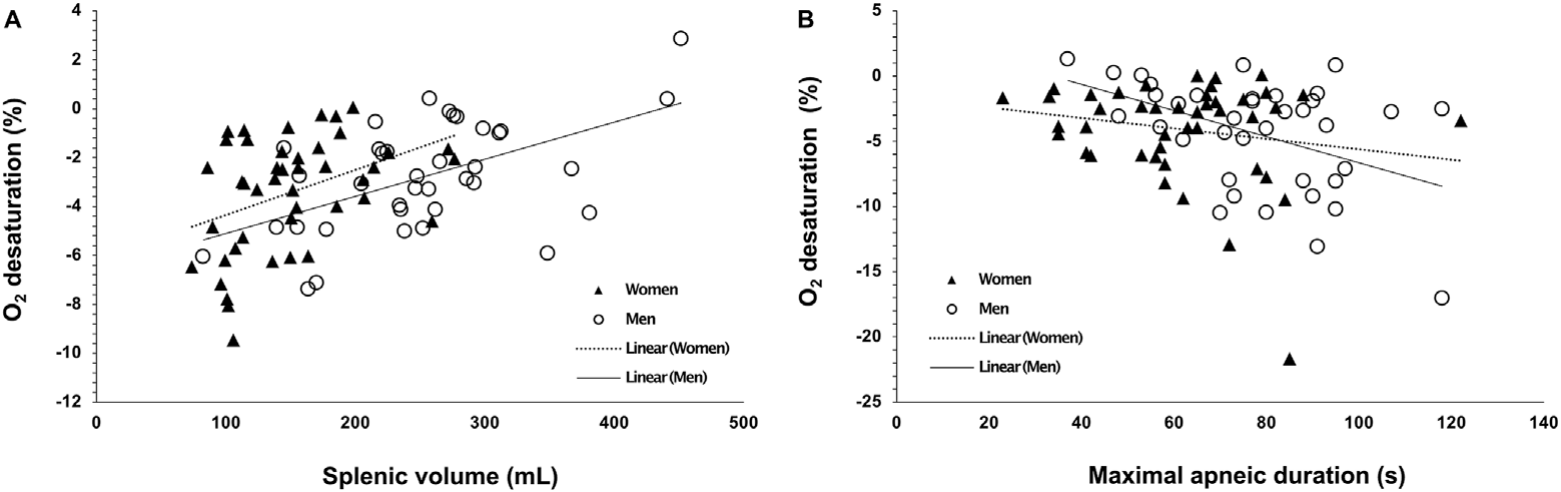
Correlation plots of **(A)** baseline (resting) spleen volume (mL) with the magnitude of oxygen desaturation (%) attained during A1, **(B)** maximal apneic duration (Apnea 2; seconds) with the magnitude of oxygen desaturation (%) attained during A2. Open circles indicate men’s data points (N = 44), and closed triangles indicate women’s data points (N = 37).

Baseline spleen volume correlated positively with O_2_ desaturation during Apnea 1 for men (r = 0.536, p < 0.001) and women (r = 0.414, p = 0.005. Figure 8A), and O_2_ desaturation attained during Apnea 2 only correlated with spleen volume for men (r = 0.472, p = 0.003), not for women (p = 0.306). The lowest SpO_2_ was positively correlated with HR reduction induced by Apnea 1 in women (r = 0.307, p = 0.043) and men (r = 0.395, p = 0.016). In addition, maximal apneic duration was negatively correlated with O_2_ desaturation attained during Apnea 2 for men (r = −0.446, p = 0.006) but not for women (p = 0.223, Figure 8B).

## 4 Discussion

This study observed a main effect on the combined dependent variables: maximal apneic duration, spleen size and contraction, HR reduction, and lung size, indicating that the model can differentiate between men and women. As far as we know, no previous research has explored the sex differences in static apnea and the related physiological responses in a combined model. This is particularly important, as these responses are physiologically interrelated. Thus, their effects should be assessed simultaneously to determine if they affect each other. There are well-known anthropometric and physiological sex differences, which may mirror enhanced exercise performance in men compared with women (1,2). Additional key findings included: (a) spleen size and absolute contraction were larger in men compared to women, while the relative contraction percentage from baseline was similar; (b) the apnea-induced HR reduction was comparable between sexes; and (c) maximal static apneic duration was longer in men than in women. These results highlight differences in physiological responses between men and women that may influence breath-holding performance. Importantly, since the study participants were non-divers with no prior training, these differences likely reflect inherent physiological characteristics rather than training-induced adaptations. Furthermore, we normalized spleen and lung volumes to body height, ensuring that the observed differences are primarily attributable to sex-related physiological factors rather than body size variations.

### 4.1 Breath-hold duration

We found that men had longer maximal apneic durations compared to women. The apneic duration of the included non-divers is consistent with previously observed apnea durations of non-divers (Schagatay et al., 2005) but substantially shorter compared with competitive freedivers (Richardson et al., 2005; Baković et al., 2005; Schagatay et al., 2012). While our investigation involved non-divers, the longer static durations in men are consistent with durations of competitive freedivers, wherein men typically exhibit ∼25% longer durations than their women counterparts (AIDA, 2024; CMAS, 2024). This may indicate that sex differences in static apnea exist across groups of different apneic dive experiences. In contrast to our findings and performances of competitive freedivers, previous laboratory investigations have failed to find longer apneic durations in men than women (Cherouveim et al., 2013; Jay and White, 2006). Cherouveim et al. (2013) measured apneic duration during two series of eight maximal apneas in air and facial immersion in water in 16 non-diving men and women. They observed no difference in maximal apneic duration between sexes. In addition, Jay and White (2006) found no difference in maximal apneic duration between sexes when matched for pulmonary capacity while performing a series of maximal duration apneas in seven different conditions. However, there are substantial methodological differences that must be considered. First, in the study by Jay and White (2006), participants were matched by pulmonary capacity, which likely resulted in more comparable apnea durations between sexes, as lung volume is a well-established determinant of apneic performance (Schagatay et al., 2012). Second, in the study by Cherouveim et al. (2013), participants performed eight maximal apneas, adding cumulative stress that could influence their performance differently. Additionally, they were tested in a supine position, which might elicit different physiological responses compared to the current study’s methodology. Nevertheless, these methodological differences across studies underscore the complexity of factors influencing breath-hold durations, including individual variability, testing environments, and protocol differences, which can make direct comparisons challenging.

The discrepancies among studies also emphasize underlying physiological differences, which highlight the inherent complexity in assessing factors that influence breath-hold durations. During brief periods of breath-holding without a drop in O_2_ levels, the physiological breaking point is mainly determined by an increase in the arterial pressure of CO_2_ (PaCO_2_), which in turn decreases the pH and stimulates central chemoreceptors, enhancing the respiratory drive (Forster et al., 2012). However, during long-duration breath-holding with hypoxemia, peripheral chemoreceptors—mainly those in the carotid bodies—increase their firing rate and further stimulate the respiratory drive. This observation indirectly suggests that individual differences in tolerance to high CO_2_ levels and low O_2_ levels could profoundly impact breath-holding duration. Possibly, the main determinant of the apnea breaking point in our study is the tolerance to high levels of CO_2_, as the participants performed relatively short durations apneas.

In addition, the brain zones where chemoreceptors are located also have sex hormone receptors (Gargaglioni et al., 2019). These become particularly important when considering sex differences to breath-holding as sex hormones change depending on the menstrual cycle. These undulating sex hormone changes subsequently influence physiological responses. A study indicate that minute ventilation, tidal volume, and peak inspiratory flow were higher in men than women in the follicular phase of the women’s menstrual cycle after brief arousal from sleep (Jordan et al., 2012). Additionally, women divers seem to have lower sensitivity to increased levels of PaCO_2_ (Peng et al., 2021), pointing out that breathing control can be very different in men and women. The maximal breath-holding breaking point is complex and determined by a variety of factors not exclusive to asphyxia (Fowler, 1954), like lung volume and diaphragm neural activity (Parkes, 2006) and psychological endurance (Schagatay, 2009). Therefore, this emphasizes the variability of maximal apneic duration and the diving response, especially in untrained non-divers (Engan et al., 2013; Costalat et al., 2017). It is plausible that our sample size may account for the significant difference in breath-holding durations observed in men, contrary to findings from other studies (Jay and White, 2006; Cherouveim et al., 2013). A larger sample size is less susceptible to the considerable individual variation typically associated with breath-holding durations.

### 4.2 The diving response

We observed that resting HR was higher in women, a common finding (Legato and Leghe, 2010; Koenig and Thayer, 2016). However, we found a similar percentage of HR reduction between sexes. Furthermore, men exhibited a lower HR nadir during time-limited apnea. Although a lower HR nadir during apnea is typically associated with better freediving performance due to enhanced O_2_ conservation (Schagatay et al., 2012), we found no relationship between HR nadir during apnea and apnea duration in either men or women. This suggests that the lower apneic HR in men may reflect their lower resting HR and does not necessarily confer any functional benefit related to O_2_ conservation. In conclusion, while baseline HR differences between men and women may lead to different apneic HRs, the overall HR reduction—indicative of the diving response—appears to be similar between the sexes.

We observed similar magnitudes of the diving response between sexes, indicating that the diving response develops equally between non-diving men and women in response to dry static apnea and is unrelated to apnea duration. However, when comparing the absolute values, women displayed higher HR at baseline and HR nadir than men. Parallel to our observations, other investigations have reported similar HR reductions in response to dry static apnea between sexes (Tocco et al., 2012; Cherouveim et al., 2013). For example, Tocco et al. (2012) observed no difference in HR reduction between sexes during dry dynamic apneas. However, they did find that men displayed a greater HR reduction during dynamic apnea with face immersion, which also resulted in similar stroke volumes and CO (Tocco et al., 2012).

Similarly, Cherouveim et al. (2013) also observed a similar degree of HR reduction between sexes in response to maximal static apneas, both dry and with facial immersion in water. Nonetheless, they also observed greater reductions in CO and increased peripheral resistance in men compared with men. Even though the diving response is frequently quantified by apnea-induced HR reduction (Schagatay and Andersson, 1998; Costalat et al., 2017; Mulder et al., 2021), the response also consists of reductions in CO and peripheral vasoconstriction (Gooden, 1994). This could imply a more pronounced diving response in men due to differences in autonomous regulation through higher sympathetic activation acting on peripheral blood vessels. However, as we did not measure CO or peripheral resistance, we can only speculate on differences in autonomous regulation between sexes to explain the observed HR responses.

### 4.3 Splenic response

We found that men had larger baseline spleen sizes than women, both in absolute terms and when normalized for height. This finding highlights that the difference is linked to sex instead of simply reflecting anatomical differences between men and women. This finding aligns with previous studies that also reported larger spleen sizes in men compared to women (Hosey et al., 2006; Ehimwenma and Tagbo, 2011; Badran et al., 2015; Chow et al., 2016; Albayrak and Server, 2019). However, other studies have found no significant differences between sexes (Prassopoulos et al., 1997). Importantly, some earlier studies did not adjust spleen size for body size, which can significantly influence results, as spleen size strongly correlates with height (Hosey et al., 2006; Chow et al., 2016). Interestingly, our study did not find an association between spleen size and height in women, suggesting that height may not be a determining factor for spleen size in women. The spleen size values in our study (women: 150 mL; men: 253 mL) fall within the normative range for healthy non-divers of both sexes, which has been reported to vary from 58 mL to 375 mL (Prassopoulos et al., 1997; De Odorico et al., 1999; Chow et al., 2016).

We found that the absolute reduction in spleen volume (splenic contraction) was greater in men than women, although the relative magnitude of the contraction was similar between sexes. This greater splenic contraction in men is a novel finding, as previous research has primarily focused on resting spleen size differences. Our results show that the volume of splenic contraction also differs between sexes in non-divers. Additionally, we observed a sex interaction effect on spleen volume changes in response to apnea, indicating that spleen volume does not change to the same extent in men and women during apnea. While the absolute magnitude of splenic contraction differed between sexes, the relative contraction (expressed as a percentage of baseline spleen size) was similar. This has also been observed in other studies where elite athletes and sedentary individuals showed significantly different absolute contractions (athletes: 46 mL vs sedentary: 30 mL) but similar relative contractions (18% vs 21%, Holmström et al., 2021). The greater absolute contraction in men is likely due to their larger spleens, which store more erythrocytes, suggesting that men experience a greater functional benefit from this contraction.

Apnea-induced splenic contraction results in a transient increase in hemoglobin concentration [Hb] by about 2%–4% (Schagatay et al., 2001; Richardson et al., 2005; Baković et al., 2005; Bouten et al., 2019). This increase in [Hb] enhances CaO_2_ and CO_2_ buffering capacity during apnea (Schagatay et al., 2001; Baković et al., 2003). However, we did not find any association between the magnitude of splenic contraction and maximal apnea duration in either men or women. This may be because participants in this study terminated their apneas before developing severe hypoxemia, which is known to trigger increased splenic contraction (Pernett et al., 2021).

### 4.4 Oxygen saturation

O_2_ desaturation was similar in men and women during the time-limited apnea but greater in men during the maximal duration apnea. However, the apnea duration was larger for men, which can explain the lower SpO_2_. We also observed a negative association between arterial O_2_ desaturation and maximal apneic duration in men but not in women, probably also related to apnea duration and the sigmoidal shape of the oxyhemoglobin dissociation curve.

Our results also highlight the significance of body O_2_ storage capacity in apneic diving. We found a positive association between VC and maximal static apneic duration in men, which was not evident in women. The reasons for this sex difference are challenging to determine. One possibility is that women may find it more difficult to extend their breath-hold times to the same extent as men, potentially due to differences in discomfort, tolerance, or mental endurance. Alternatively, it could reflect an underlying physiological mechanism that causes women to rely on other compensatory factors to a greater extent than men rather than solely depending on vital capacity. This may suggest a divergence in strategies for maintaining oxygenation during apnea. Associations between VC and maximal apnea time are in line with previous research exhibiting greater lungs in competitive freedivers that relate positively to their freediving performance (Schagatay et al., 2012) and that a larger percentage of forced VC is associated with increased breath-hold diving performance (Peng et al., 2021). Additionally, our finding that men demonstrate greater height-adjusted lung volumes aligns with prior research (e.g., Schwartz et al., 1988). This suggests that the differences in lung volumes between men and women cannot be solely attributed to anatomical differences or variations in body size. Instead, these findings point to a physiological basis, potentially linked to sex-specific adjustments or intrinsic differences in lung structure and function. The lungs are the body’s primary O_2_ reservoir, reflecting a substantial anatomical advantage in men compared with women.

### 4.5 Limitations

There are a few limitations in our data collection that should be acknowledged. First, we did not consider the menstrual cycle phase of the female participants, which can affect ventilatory drive and cellular metabolism due to hormonal fluctuations in progesterone and estrogen levels (Cherouveim et al., 2020). This could impact maximal apnea duration. However, since we collected a random sample, we likely had female participants at different stages of the menstrual cycle, which balanced out the individual effects of each phase to some extent. Future studies could track menstrual cycle phases to understand these phase-specific effects better.

Second, no blood samples were taken during the apnea tests, which prevents us from directly linking splenic contraction to increased hemoglobin concentration [Hb] and the associated functional benefits on CaO_2_. We believe that spleen volume reduction in response to apnea could potentially correlate with a change in [Hb] for two main reasons. First, a multitude of laboratory studies have reliably confirmed that transient splenic contraction is closely associated with transiently elevated [Hb] in a variety of environments (Schagatay et al., 2005; 2007; Bouten et al., 2019; Bakovic et al., 2003; Bakovic et al., 2005). Second, recent studies comparing splenectomized individuals with those with intact spleens prove that human splenic contraction occurs during apnea, resulting in elevated [Hb]. This response is absent in splenectomized individuals (Schagatay et al., 2001; Bakovic et al., 2003; Agostoni et al., 2005; Baković et al., 2005).

Since our participants were not experienced divers, they might have found it challenging to hold their breath for as long as possible. They could have stopped the breath-holding due to discomfort. We also did not measure involuntary breathing movements, making the determination of the different stages of breath-holding challenging. As a result, we could not be firm that our participants reached their physical limits, so we could not rule out physical endurance as a limiting factor for both women and men. However, we standardized the breath-holding test for both men and women to ensure comparable results.

The results may have been influenced by caffeine, as our protocol required participants to abstain from caffeine-containing beverages for only 1 h before arriving at the laboratory. Given that the half-life of caffeine ranges from 2.5 to 4.5 h (Arnaud, 1987), 1 h may not be sufficient for the caffeine’s effects to dissipate. Additionally, we did not examine potential differences between sexes regarding caffeine intake, as metabolism may vary between individuals (Nehlig, 2018).

## 5 Conclusion

We found that men with no prior experience in apnea diving had larger spleens, greater splenic contraction, larger lung volumes, and longer maximal apnea durations than women. Additionally, the magnitude of the diving response, as indicated by the apnea-induced HR reduction, was similar between the sexes. The longer breath-hold duration observed in men may be partly attributed to their greater capacity for O_2_ storage, particularly in the lungs and, to a lesser extent, in the spleen, contributing to increased arterial O_2_ availability through splenic contraction. Thus, combining larger spleens and greater lung capacity may explain the sex differences in breath-hold duration. Although men and women exhibit similar cardiovascular responses, the larger O_2_ stores in men likely account for their superior performance in breath-holding.

## Data Availability

The raw data supporting the conclusions of this article will be made available by the authors, without undue reservation.
